# Incidental Thyroid Carcinoma Diagnosed after Total Thyroidectomy for Benign Thyroid Diseases: Incidence and Association with Thyroid Disease Type and Laboratory Markers

**DOI:** 10.1155/2013/451959

**Published:** 2013-11-20

**Authors:** D. Askitis, E. I. Efremidou, M. Karanikas, A. Mitrakas, G. Tripsianis, A. Polychronidis, N. Liratzopoulos

**Affiliations:** First Surgical Department, Medical School, University General Hospital of Alexandroupolis, Democritus University of Thrace, University Campus Dragana 1, 68100 Alexandroupolis, Greece

## Abstract

*Objective*. Currently, total thyroidectomy (TT) is widely used to treat benign thyroid diseases and thyroid carcinoma. The differential diagnosis between benign and malignant thyroid disorders and the potential identification of thyroid microcarcinomas with biochemical markers remain controversial. This retrospective study aimed to estimate the prognostic validity of thyroid autoantibodies, thyroglobulin (Tg), and the thyroid disease type in diagnostic approaches regarding the co-existence of incidental thyroid carcinoma (ITC) with benign thyroid diseases. *Methods*. A cohort of 228 patients was treated with TT for benign thyroid disorders between 2005 and 2010. Thyroid autoantibodies and Tg were preoperatively estimated. Patients were classified according to the preoperative and histologically established diagnoses, and the median values of the biochemical markers were compared between the groups. *Results*. ITC was detected in 33/228 patients and almost exclusively in the presence of nontoxic thyroid disorders (*P* = 0.014). There were no statistically significant differences in the median values of the biochemical markers between the benign and malignant groups. There was also no significant association between ITC and chronic lymphocytic thyroiditis. *Conclusions*. The co-existence of ITC with benign and especially nontoxic thyroid diseases is significant, and treatment of these disorders with TT when indicated can lead to the identification and definitive cure of microcarcinomas. Further studies are required to establish precise markers with prognostic validity for TC diagnosis.

## 1. Introduction

Thyroid diseases are very common in clinical practice, and their diagnosis and treatment are based upon detailed assessments that are aimed at achieving optimal therapeutic outcomes. Medical history, clinical examination, laboratory tests, and imaging findings are essential for their diagnosis and also for determining whether surgical treatment is indicated, subsequent to an evaluation and consultation with an endocrinologist [1, 2].

The therapeutic strategies for thyroid diseases include the following:

1. Follow-up with regular assessments of thyroid function and morphology, especially in patients with solitary or multiple thyroid nodules.
2. Medical treatment for any thyroid pathology associated with hyper- or hypothyroidism for achieving euthyroidism and symptom improvement.
3. Interventional treatment with radioiodine ablation in cases of hyperthyroidism when medical therapy fails to achieve an appropriate clinical recovery, and thyroidectomy is contraindicated or not accepted by the patient.
4. Indication for surgical treatment if malignancy is detected or otherwise suspected.

Surgical treatments include subtotal or near-total thyroidectomy for preserving functioning thyroid tissue and minimizing the possibility of complications and total thyroidectomy (TT), which is the optimal therapy for thyroid carcinoma (TC).

The use of TT remains controversial for treating small differentiated thyroid carcinomas (DTCs) and even more for treating benign diseases [3–5].

Thus, many surgeons avoid TT due to possible severe complications such as permanent recurrent laryngeal nerve palsy and permanent hypoparathyroidism [3–5].

Although the extent of resection for benign diseases remains under debate, currently an increasing number of total thyroidectomies have been performed in specialist endocrine surgery units, and indications for TT include Graves' disease (GD) and multinodular goiter. Furthermore, the complication rates of permanent recurrent laryngeal nerve palsy (0–1.3%) and permanent hypoparathyroidism (1%) following subtotal thyroidectomy were found to be similar to those after TT [6, 7].

A further advantage of TT is the definitive removal of incidental small DTC. The prevalence of thyroid incidentalomas is high in the general population (13–67%), and although the majority of these are benign, the small percentage of occult carcinomas cannot be diagnosed with routine imaging methodologies or the gold standard diagnostic technique of fine needle aspiration biopsy (FNAB) [8, 9]. It remains controversial whether some standard laboratory tests might be useful in identifying malignancy in patients with these incidentalomas.

The main aim of the present retrospective study is to investigate whether the thyroid disease type and specific biochemical parameters such as thyroid autoantibodies-thyroid peroxidase antibodies (TPOAbs) and thyroglobulin antibodies (TgAbs-) and thyroglobulin (Tg) levels correlate with the presence of incidental TC. The potential validity grade might present a useful diagnostic tool for establishing more accurate therapies with regard to indications for thyroidectomy.

## 2. Materials and Methods

The retrospective study included a total cohort of 228 (182 females and 46 males) patients with a mean age of 49.60 ± 14.65 years who were treated with TT for various benign thyroid diseases between January 2005 and March 2010.

All the patients were of European Caucasian ethnicity and lived in the geographical zones from East Macedonia to Thrace in Northern Greece. Most patients (217, 95.2%) were referred by an endocrinologist or family physician, whereas 11 (4.8%) patients came directly to the clinic for surgical consultations.

Data on sex, age, thyroid disease type, medications, preoperative laboratory and imaging assessments, and final pathology were extracted from the hospital medical records.

Indications for surgery were established after a detailed clinical, laboratory, and imaging evaluation. Major indications for surgery in patients with euthyroidism included severe local symptoms such as a mechanical obstruction due to a large goiter or multiple nodules in both lobes. The most common indication for surgery in patients with functioning thyroid disease was disease relapse either after discontinuation of the pharmacological therapy or previous radioiodine application with progressive general manifestations, in some cases with additional pressure symptoms. The patients with hyperthyroid diseases had been treated with antithyroid agents for a mean period of 25.4 months. Additional indications for TT in these subjects included the refusal of radioactive iodine treatment in 20% cases because patients feared exposure to radiation and/or wanted a rapid and permanent solution for their symptoms.

Exclusion criteria included any type of previous thyroidectomy, a history of head and/or neck radiation, a clinical or cytological suspicion or diagnosed thyroid malignancy, and a cytological diagnosis of autoimmune thyroiditis.

All thyrotoxic patients received pharmacologic therapy, which was continued until the day of the surgery, to prevent perioperative thyroid crisis.

Thyrotropin (TSH), thyroxin (*T*_4_), triiodothyronine (*T*_3_), TPOAbs, TgAbs, and Tg serum levels were evaluated in all patients. The reference values used in our Hormone-Immunology Department were as follows: *T*_4_: 4.0–11.0 *μ*g/dL; *T*_3_: 0.7–1.8 ng/mL; TSH: 0.38–3.8 *μ*IU/mL; Tg-Abs: 0–120 ng/mL; TPO-Abs: 0–34 U/mL; and Tg: 1.4–78 ng/mL.

All subjects were simultaneously assessed by thyroid ultrasound and scintigraphy at the University Department of Nuclear Medicine. Preoperative diagnoses were determined on the basis of functional and morphological criteria, and the patients were classified into the following groups: (1) goiter with solitary nonfunctioning thyroid nodule (STN) group, (2) nontoxic multinodular goiter (NTMG) group, (3) toxic multinodular goiter (TMG) group, (4) Toxic adenoma (TA) group, and (5) Graves' disease (GD) group.

To exclude preexisting vocal cord palsy, an otolaryngologist assessed the preoperative vocal cord motility in all patients.

All patients in a euthyroid state underwent elective classic TT. Surgical dissection of the thyroid was performed after the identification and preservation of both laryngeal nerves and the superior and inferior parathyroid glands. In all patients, 3-4 parathyroid glands were identified and were left *in situ* with their own vascularization. If a parathyroid gland was inadvertently excised or devascularized, we autotransplanted the gland into the ipsilateral sternocleidomastoid muscle. We drained the neck with bilateral suction drains for 48 hours, and the patients were usually discharged within 4 days after surgery.

The surgical specimens were histologically examined at the University Pathology Department.

Written informed consent was obtained from all patients, and the regional ethical committee approved the study.

Statistical analyses of the data were performed using the Statistical Package for the Social Sciences (SPSS), version 19.0 (IBM). The normality of quantitative variables was tested using the Kolmogorov-Smirnov test. The levels of biochemical markers were expressed as the median and interquartile range (IQR). The chi-square test and the odd ratios (OR) with their 95% confidence intervals (CI), calculated by means of simple logistic regression analysis, were used to evaluate any potential associations between the categorical variables. The Mann-Whitney *U*-test and the Kruskal-Wallis test were used to assess differences in biochemical markers between two or more patients groups, respectively. All tests were two tailed, and statistical significance was defined as a *P* value less than 0.05.

## 3. Results

The 228 patients in the study cohort were aged between 11 and 80 years, with a median age of 50 years (IQR, 39 to 61 years). This cohort comprised 182 female patients (79.8%; median age, 48 years) and 46 male patients (20.2%; median age, 56 years). The preoperative diagnoses were as follows: euthyroid solitary nodule within a goiter (*n* = 34, 14.9%), NTMG (*n* = 152, 66.7%), TMG (*n* = 26, 11.4%), toxic adenoma (*n* = 6, 2.6%), and GD (*n* = 10, 4.4%). The median thyroid volumes assessed by ultrasound were as follows: NTMG 78.7 cm^3^ (52.5–115.2); STN 62.6 cm^3^ (48.4–87.3); TMG 71.8 cm^3^ (47.7–106.0); goiter with toxic adenoma 50.7 cm^3^ (31.6–60.2); and Graves' disease 56.4 cm^3^ (38.6–71.3) (*P* = 0.608).

The histological analysis of the surgical specimens indicated that 33 cases (14.5%) had TC, while 195 (85.5%) cases did not have any malignant lesion. All detected TCs were ≤1 cm in diameter.

The patients who were diagnosed with malignancies were categorized into the following subgroups: 31 (93.9%) were classified as having DTC; of these, 27 (81.8%) were classified as having papillary TC and 4 (12.1%) as having follicular TC. According to the 7th edition of TNM classification for thyroid cancer adopted by the International Union Against Cancer, 26 of the papillary TCs were classified as T1aN0M0 and 1 as T1aN1M0. All 4 patients with follicular TC were categorized as T1aN0M0. One (3%) patient was found to have medullary (T1aN1M0), and 1 (3%) anaplastic carcinoma (T4aN0M0). The female/male patient ratio was 22/11 or 2 : 1. The incidence of carcinoma varied significantly among the 5 preoperative diagnosis groups (*P* = 0.003) (Table 1). Nearly one-third of the patients (11/34 or 32.4%) with nontoxic solitary nodules and nearly 14% (21/152) of the patients with euthyroid multinodular goiters were ultimately diagnosed with malignant thyroid incidentaloma. The correlation of histologically diagnosed TC with the preoperatively evaluated solitary nodule in the STN group was 63.6% (7/11). In the patient group with autonomous thyroid diseases, only 1 young male patient with GD had a coexisting papillary thyroid microcarcinoma. This patient represented 10% of the GD group and 2.4% of all individuals with hyperthyroidism (1/42). The prevalence of thyroid cancer in the group with euthyroid diseases was 17.2% (32/186; *P* = 0.014, odds ratio (OR): 8.5, 95% CI: 1.1–64.2).

The thyroid specimens from 56 patients (24.6%) had histopathological features of chronic lymphocytic thyroiditis (CLT), and 172 (75.4%) showed no histological evidence of chronic inflammation. Histological criteria for the diagnosis were the presence of diffuse infiltrations from T- and B-lymphocytes with destruction of the physiological architecture of thyroid follicles and formation of lymphoid follicles and germinal centers and multiple Hürthle cells. The incidence rate of CLT was similar between the different preoperative diagnosis groups (*P* = 0.946) (Table 1).

The patients in whom TC was identified were classified into 2 subgroups according to the concurrence of CLT. Six of the 33 patients (18.2%) with TC presented with coexistent elements of CLT. A further classification showed that 6 of the 31 patients (19.3%) with DTC presented with concomitant CLT, whereas the prevalence of CLT in patients without DTC was 25.6% (*P* = 0.451, OR: 1.4, 95% CI: 0.6–3.7). In all, 10.7% of the patients with CLT developed DTC, whereas 14.5% of the patients without CLT were diagnosed with DTC (*P* = 0.477, OR: 1.4, 95% CI: 0.6–3.6).

We compared the median values of the biochemical markers that were estimated preoperatively in all groups of patients (Table 2). Twenty-nine patients were excluded from the evaluation of the median Tg values due to the presence of high TgAbs titers above the upper laboratory reference rate and the high probability of false high or low Tg levels [10]. Statistically significant variations were found for Tg (*P* = 0.002) and age (*P* < 0.001).

Moreover, Tg values were considerably elevated in the hyperthyroid disease patient subgroups in comparison to those without thyroid autonomy (162.65 (63.01–418.91) versus 52.80 (18.50–151.10); *P* < 0.001). TPOAb median titers were elevated above the upper reference level only in the GD group (median = 63.55), whereas TgAb median levels were within the reference range in both euthyroid and autonomous thyroid disease patients. Another interesting aspect was the very low median age of 29 years observed in the GD patients compared to the other 4 groups (median age 48.50 years (39.00–60.75); *P* = 0.004).

The median values of the preoperatively estimated biochemical parameters were also measured in the histologically established benignity and malignancy groups (Table 3). There were no statistically significant differences between the median levels of the biochemical markers with regard to benignity and thyroid cancer, both overall and within the male and female subgroups. The median titers of the thyroid autoantibodies were evaluated as follows: TgAbs, 21.40 versus 20.00 in patients with and without thyroid malignancy (*P* = 0.657) and TPOAbs, 10.35 versus 12.60 in patients with and without thyroid malignancy (*P* = 0.329), respectively. Median Tg levels were similar in both subgroups (62.75 versus 58.0 in patients with and without thyroid malignancy; *P* = 0.696). All median values were also within the laboratory reference ranges. The only exception was the Tg level in male patients with thyroid cancer, which was considerably elevated but was not statistically significant when compared to the group of men without malignancy (*P* = 0.169). The median age of patients with incidental TC was also similar to that of patients without malignancy (48 years versus 50 years, *P* = 0.785).

A statistical analysis was performed for the solitary thyroid nodule and NTMG subgroups that included most of the identified malignancies (Table 3). Patients with NTMG and TC presented with Tg levels above the upper reference range when compared to patients without TC, but this difference did not reach statistical significance (*P* = 0.380). Differences in the median thyroid autoantibody titers also failed to achieve statistical significance in these groups.

The patients with thyroid cancer were arranged into 2 subgroups based on the concurrence of CLT, and the preoperative median biochemical marker levels were evaluated in both groups (Table 4(a)). The thyroid autoantibody levels were significantly elevated in the TC + CLT group (TgAbs: 53.65 versus 14.70, *P* = 0.039; TPOAbs: 103.90 versus 6.25, *P* = 0.011). TPOAbs were also elevated above the upper reference range. The median Tg value was above the reference range in the patient group with malignancy and without thyroiditis, but the difference in comparison to the TC + CLT group was not statistically significant (*P* = 0.287).

A respective evaluation of the biochemical markers was performed in the group of 50 patients with thyroiditis and without concomitant TC, compared to the group of 6 patients with concomitant thyroiditis and TC (Table 4(b)). No statistically significant differences in the median laboratory values were observed between the 2 groups. TPOAbs levels were similarly elevated above the reference range in both groups (*P* = 0.826), whereas TgAbs and Tg levels were within the normal range in both groups (*P* = 0.341 and *P* = 0.749, resp.).

## 4. Discussion

Thyroid diseases are mainly benign and can be treated conservatively. The diagnostic approach to thyroid disorders and especially the differential diagnosis between benign and malignant lesions is challenging for physicians and is based upon a combination of clinical, imaging, and laboratory parameters.

Thyroid autoantibody levels are evaluated to determine the autoimmune background in several thyroid diseases; Tg levels can be used as a marker in the follow-up of patients with DTC who underwent TT after Tg secretion is stimulated by the withdrawal of l-thyroxin postoperative substitution therapy or by rhTSH administration [11, 12]. Tg has also been proposed as a predictive marker for differentiated thyroid cancer manifestation, especially for follicular nodules with indeterminate cytology [13]. Although autoimmune thyroid diseases, especially Hashimoto's thyroiditis, have been related to thyroid cancer, only the relationship between these diseases and thyroid lymphoma is well established [14].

TC is the most common endocrine carcinoma, as it accounts for almost 90% of all endocrine malignancies. During the last 30 years, the rate of TC incidence has increased worldwide, with average increases of 48.0% in men and 66.7% in women [15]. Key points in the diagnostic algorithm for TC include suspicious characteristics in imaging analyses, such as solid nodules, microcalcifications, high internal blood flow as determined by Doppler imaging, hypoechogenicity and irregular borders in thyroid ultrasonography, low radionuclide uptake in scintigraphy, as well as positive and/or suspicious FNAB [16]. FNAB has a mean sensitivity of 83%, a specificity of 92%, and a positive predictive value of 75%. The mean false-positive and false-negative rates of the method are reported as <5% [2]. The main limitations of FNAB are the inability of cytology to distinguish follicular and Hurthle-cell carcinomas from the respective benign adenomas, the requirement of an experienced prober to avoid nondiagnostic punctures, and the presence of multiple nodules in a goiter [17, 18].

TT is considered the most effective method for achieving complete thyroid malignancy cures [19]. TT has shown a similar or even lower incidence of postoperative complications in comparison to partial or subtotal thyroidectomy. Furthermore, TT is associated with a lower rate of disease recurrence. TT also features the advantages of histological establishment and the permanent cure of nondiagnosed DTC. These small malignancies have been reported in significant percentages of cases (5.0–22.0%) from various surgical studies; therefore, a preoperative indication of small DTC existence would be useful [20–23].

Taken together, the above findings motivated the present study to investigate the possible predictive value of thyroid autoantibodies, Tg, and thyroid disease type in diagnostic evaluations for occult malignancy in patients treated with TT for benign thyroid disorders. The hypothesis would be of interest if statistically significant preoperative relationships between the above-mentioned biochemical markers, thyroid disease type, and thyroid malignancy could be detected in patients treated with TT for benign thyroid diseases. The comparison was performed between patients with histological diagnoses of incidental thyroid cancer versus those without TC, according to final pathology. Any significant association could provide assistance in decisions of either conservative medical treatment or surgical therapy.

The study limitations included the small size of specific patient subgroups, especially those with hyperthyroidism, as well as the local origin of all the patients. The sample of histologically detected incidental carcinomas and chronic lymphocytic thyroiditis was also small and this parameter could conceivably influence the grade of statistical significance when comparing the median laboratory levels between benign and malignant groups. Another limitation was the use of thyroid-specified drugs, especially in patients with hyperthyroidism, although no evidence exists that this parameter can affect the titers of the examined markers.

The total prevalence of incidental TCs in the study cohort was 14.5% (33/228 patients), with a variable distribution in the different study groups; this difference was statistically significant (*P* = 0.003). Patients with autonomous thyroid disorders were all free of malignancy, except for 1 patient who was diagnosed with an incidental papillary carcinoma during final pathology. The prevalence of thyroid malignancy was 8.5 times higher in patients without hyperthyroidism.

Several studies reported a lower incidence of thyroid malignancy in patients with hyperthyroidism versus those with non-toxic goiters. The exception is the development of a solitary nodule in coexisting GD. Gabriele et al. reported a 1.65% rate of differentiated thyroid cancer in 425 patients with hyperthyroidism, while no DTC was observed in final pathology analyses after TT for the treatment of GD [24]. The retrospective study conducted by Phitayakorn and McHenry also reported a very low incidence of DTC (2/93 TT) in the presence of GD [25]. Another clinical study from Spain reported a much higher incidence of DTC that was unrelated to age in patients with euthyroid multinodular goiters versus those with GD [26]. According to Ardito et al., the prevalence of DTC was 5.6% in a total of 408 patients who were treated with thyroidectomy for autonomous thyroid disorders [27]. Belfiore et al. reported more aggressive thyroid cancer behavior in GD patients compared to those with toxic adenoma and nonfunctioning thyroid disorders, but this finding was not supported by a Japanese study including 154 patients with GD and concomitant papillary carcinoma [28, 29].

Taking into account the limitation of the small sample size of hyperthyroid patients, the results in the current study suggest the hypothesis that autonomous thyroid function may have a protective role against the development of thyroid cancer. Molecular research on biological markers may detect a potential inhibitory role in the development of thyroid malignancy. The suppression of thyrotropin stimulatory action, particularly on thyrocytes with genetic mutations that predispose the cells to nodular formation and malignancy, could be a possible mechanism.

Moreover, in the current study, the comparison between groups of patients with incidental carcinoma and those without malignancy in pathology did not reveal any statistically significant differences in the preoperative median values of thyroid autoantibodies or Tg, either in the total cohort or in gender and preoperative diagnosis subgroups. Zimny et al. examined 119 follicular TC cases and indicated that a Tg value > 300 ng/mL could be used as an optimal cutoff value in the differentiation of benign and malignant nodules and proposed Tg levels as an independent risk factor for malignancy [30]. Similarly, Petric et al. examined 388 follicular TCs to investigate potential independent predictive factors for TC and found that the combination of preoperative Tg levels > 400 ng/mL and age < 45 years might be predictive in patients with solitary nonfunctional thyroid nodules [31].

Suh et al. conducted a study to distinguish preoperatively follicular and Hurthle carcinomas from respective adenomas by Tg assessment in a total of 39 patients; however, the results of this study did not establish a significant difference in Tg values between patients with benign and malignant nodules [32]. Kim et al. also evaluated 1638 patients with thyroid nodules and concluded that the prevalence of TC was higher in patients with high preoperative titers of TgAbs, whereas there was no correlation between high TPOAbs and TC [33]. Another recent study reported an increased risk of suspicious or malignant cytology when FNAB was performed on solitary thyroid nodules in patients with positive thyroid antibody levels. However, patients with thyroid nodules and cytologically indeterminate risk who were histologically diagnosed with carcinoma after TT did not have positive thyroid antibody levels [34]. Thus, the use of the above markers as diagnostic tools for thyroid malignancy remains under discussion.

The fact that the chronic inflammatory process is an established predisposing factor for malignant alteration has motivated many studies that have tried to find an association between autoimmune thyroid disease and an elevated risk of cancer manifestation. In the present study, there was no statistical significance in DTC development between patients with CLT and those without CLT (*P* = 0.477). Furthermore, no significant difference was detected between patients with CLT + benignity and those with CLT + TC (*P* = 0.451). Thus, CLT does not appear to be an independent risk factor for malignant alteration in the thyroid gland. This conclusion seems to be in accordance with that of Costanzo et al., who reported that no clear statistical significance was present in the coexistence of CLT and DTC in cytologic material [35]. A study in Croatia of 10,508 patients with thyroid nodules who underwent FNAB reported similar results [36]. In contrast, another study concluded that the higher incidence of TC or suspicious thyroid lesions in patients with thyroid nodules is associated with elevated levels of thyroid autoantibodies and TSH, as well as concurrent CLT. An interesting feature of this study is the lower incidence of DTC diagnosed by FNAB in nodules of uncertain malignancy among patients with positive thyroid antibody titers, compared to those with negative antibody titers [37]. This finding might also bring into question the validity of those markers as prognostic factors for the development of thyroid cancer.

Kim et al. performed a retrospective study of 1329 patients in Korea who underwent TT for benign and malignant thyroid diseases and found strong correlations between CLT and papillary TC or multifocused DTC, although there were no correlations between CLT and small tumors or metastatic disease [38]. Similar results were reported by Ahn et al. who indicated a high incidence of concomitant CLT and aggressive thyroid cancer [39]. Although there were no observed significant differences in patients with CLT associated with DTC compared to those with CLT alone in the present study, the high prevalence of CLT in the general population indicates that a careful follow-up is essential. Further clinical studies are needed to investigate the possible association between CLT and TC development.

In the current study, there was no statistically proven prognostic value of thyroid autoantibody and Tg levels for TC. Nevertheless, it is interesting that thyroid microcarcinoma cases were doubled in female patients, compared to male patients. It is also clear that incidental DTC occurs frequently in patients with a solitary thyroid nodule, whereas the prevalence of DTC is minimal in cases with hyperthyroidism. In addition, this study does not reveal a statistically significant association of CLT with TC.

Further, the results of the Tg investigation do not seem to justify a predictive value for preoperative diagnosis or suspicion of coexisting TC in benign thyroid disease, although the Tg level seemed to be associated with autonomous thyroid function. This finding was incidental during the study and since there are no reported clinical studies that investigated the potential role of Tg in the monitoring of toxic thyroid disorders, this might introduce a new research field. Future clinical studies must be performed to determine precise laboratory or other markers for the preoperative diagnosis of thyroid cancer.

Finally, authors should indicate the significant prevalence of thyroid microcarcinomas among benign thyroid diseases; this prevalence would suggest TT as the preferred method in benign thyroid diseases for which surgery is indicated. The choice of TT in such cases is justified, as TT achieves a permanent cure of thyroid disorders and a definitive removal of possible concomitant thyroid malignancy with a low rate of postoperative complications or disease recurrence.

## Figures and Tables

**Table 1:** Arrangement of patients after preoperative diagnosis and co-existence of thyroid cancer and CLT.

Diagnosis	No. of patients	TC	CLT
STN	34	11 (32.4)	7 (20.6)
NTMG	152	21 (13.8)	38 (25.0)
TMG	26	0 (0.0)	7 (26.9)
*Τ*oxic adenoma	6	0 (0.0)	2 (33.3)
Graves' disease	10	1 (10.0)	2 (20.0)

Total	228	33 (14.5)	56 (24.6)

TC: thyroid cancer; CLT: chronic lymphocytic thyroiditis; STN: nontoxic solitary thyroid nodule; NTMG: nontoxic multinodular goiter; TMG: toxic multinodular goiter.

Data were expressed as frequencies and percentages (%).

**Table 2:** Biochemical markers (expressed as median values and interquartile range) in relation to the preoperative diagnosis.

	STN (*n* = 34)	NTMG (*n* = 152)	TMG (*n* = 26)	*Τ*oxic adenoma (*n* = 6)	Graves (*n* = 10)	*P* value
TgAbs	21.45 (12.75–38.15)	19.75 (10.00–61.55)	16.95 (10.00–76.68)	28.70 (24.60–743.35)	90.05 (27.79–1421.88)	0.231
TPOAbs	14.00 (2.00–31.95)	11.85 (4.00–21.20)	11.36 (7.77–84.10)	20.70 (10.93–196.70)	63.55 (10.90–1371.33)	0.081
Tg	36.90 (13.98–136.72)	58.00 (20.30–155.30)	182.30 (113.5–455.85)	117.50 (28.35–214.30)	211.37 (44.77–1150.5)	0.002
Age	42 (28–58)	50 (40–61)	62 (49–71)	50 (39–54)	29 (24–47)	<0.001

TgAbs (ng/mL); TPOAbs (U/mL); Tg (ng/mL); Age (years); STN: nontoxic solitary thyroid nodule; NTMG: nontoxic multinodular goiter; TMG: toxic multinodular goiter; *P* value, Kruskal-Wallis test.

**Table 3:** Biochemical markers (expressed as median values and interquartile range) in relation to the presence of carcinoma, patients' gender, and preoperative diagnosis.

	TC		*P* value
	No	Yes	
Total			
TgAbs (ng/mL)	20.00 (10.00–41.90)	21.40 (12.25–91.66)	0.657
TPOAbs (U/mL)	12.60 (5.15–29.65)	10.35 (2.00–33.96)	0.329
Tg (ng/mL)	58.00 (20.52–194.00)	62.75 (21.43–421.5)	0.696
Age (years)	50 (40–61)	48 (38–61)	0.785
Females			
TgAbs (ng/mL)	21.38 (10.33–85.48)	23.00 (12.50–89.40)	0.695
TPOAbs (U/mL)	13.00 (5.00–36.34)	8.70 (3.25–24.23)	0.274
Tg (ng/mL)	57.75 (24.10–209.82)	60.70 (18.55–204.80)	0.715
Age (years)	50 (40–60)	47 (37–60)	0.623
Males			
TgAbs (ng/mL)	14.68 (9.00–29.41)	14.60 (8.40–1224.10)	0.860
TPOAbs (U/mL)	10.98 (5.65–17.15)	17.70 (1.50–102.35)	0.875
Tg (ng/mL)	64.30 (12.40–146.90)	216.80 (25.80–571.02)	0.169
Age (years)	55 (41–63)	60 (37–65)	0.793
Solitary nodule			
TgAbs (ng/mL)	21.37 (9.75–33.20)	26.95 (14.60–98.45)	0.364
TPOAbs (U/mL)	11.25 (3.67–21.04)	18.00 (2.00–91.95)	0.352
Tg (ng/mL)	27.00 (12.71–79.17)	45.20 (24.08–503.57)	0.253
Age (years)	42 (28–55)	50 (27–66)	0.422
NTMG			
TgAbs (ng/mL)	19.80 (10.00–41.25)	18.20 (10.00–89.40)	0.966
TPOAbs (U/mL)	12.30 (4.18–24.85)	6.25 (2.12–15.98)	0.213
Tg (ng/mL)	56.60 (20.52–142.45)	92.92 (16.75–461.35)	0.380
Age (years)	50 (40–61)	48 (39–61)	0.751

TC: thyroid cancer; NTMG: nontoxic multinodular goiter; *P* value, Mann-Whitney *U*-test.

**Table:** (a) Biochemical markers (expressed as median values and interquartile range) in the subgroups of patients with TC alone and TC + CLT.

	TC alone	TC + CLT	*P* value
TgAbs (ng/mL)	14.70 (11.75–27.68)	53.65 (25.96–91.66)	0.039
TPOAbs (U/mL)	6.25 (2.00–15.97)	103.90 (25.91–172.40)	0.011
Tg (ng/mL)	92.35 (20.75–497.50)	47.00 (17.67–132.75)	0.287
Age (years)	47.00 (34.50–60.50)	56.50 (40.50–65.50)	0.250

TC: thyroid cancer; CLT: chronic lymphocytic thyroiditis; *P* value, Mann-Whitney *U*-test.

**Table:** (b) Biochemical markers (expressed as median values and interquartile range) in the subgroups CLT alone and TC + CLT.

	CLT alone	TC + CLT	*P* value
TgAbs (ng/mL)	105.40 (23.00–400.60)	53.65 (25.96–91.66)	0.341
TPOAbs (U/mL)	75.62 (13.21–495.84)	103.90 (25.91–172.40)	0.826
Tg (ng/mL)	42.15 (13.01–83.65)	47.00 (17.67–132.75)	0.749
Age (years)	46.00 (37.50–57.00)	56.50 (40.50–65.50)	0.320

TC: thyroid cancer, CLT: chronic lymphocytic thyroiditis; *P* value, Mann-Whitney *U*-test.
